# Dihydromyricetin improves social isolation-induced cognitive impairments and astrocytic changes in mice

**DOI:** 10.1038/s41598-022-09814-5

**Published:** 2022-04-07

**Authors:** Saki Watanabe, Alzahra Al Omran, Amy S. Shao, Chen Xue, Zeyu Zhang, Jifeng Zhang, Daryl L. Davies, Xuesi M. Shao, Junji Watanabe, Jing Liang

**Affiliations:** 1 Titus Family Department of Clinical Pharmacy, School of Pharmacy, University of Southern California, 1985 Zonal Ave, PSC 504, Los Angeles, CA 90033 USA; 2 Homer Stryker M.D. School of Medicine, Western Michigan University, Kalamazoo, MI 49007 USA; 3 Translational Research Laboratory, School of Pharmacy, University of Southern California, Los Angeles, CA 90033 USA; 4 David Geffen School of Medicine, University of California Los Angeles, Los Angeles, CA 90095 USA

**Keywords:** Cellular neuroscience, Cognitive neuroscience, Glial biology, Learning and memory, Neuroimmunology, Stress and resilience, Anxiety

## Abstract

Social isolation induces stress, anxiety, and mild cognitive impairment that could progress towards irreversible brain damage. A probable player in the mechanism of social isolation-induced anxiety is astrocytes, specialized glial cells that support proper brain function. Using a social isolation mouse model, we observed worsened cognitive and memory abilities with reductions of Object Recognition Index (ORI) in novel object recognition test and Recognition Index (RI) in novel context recognition test. Social isolation also increased astrocyte density, reduced astrocyte size with shorter branches, and reduced morphological complexity in the hippocampus. Dihydromyricetin, a flavonoid that we previously demonstrated to have anxiolytic properties, improved memory/cognition and restored astrocyte plasticity in these mice. Our study indicates astrocytic involvement in social isolation-induced cognitive impairment as well as anxiety and suggest dihydromyricetin as an early-stage intervention against anxiety, cognitive impairment, and potential permanent brain damage.

## Introduction

Anxiety disorders are commonly regarded as a precursor for mild cognitive decline^1^. The COVID-19 pandemic and its associated fear and stress have inflicted a great burden on the mental wellbeing of many individuals, especially as millions have lost their loved ones^2^. Anxiety disorder prevalence is predicted to continue growing in the post-pandemic period^3^. Notably, social isolation has been the norm for many during the pandemic, and several studies have demonstrated its negative impact on mental wellbeing, including reports of acute stress disorder (ASD) along with depression, anxiety, and insomnia^4–6^. Its consequences are suggested to increase the risk of acute or chronic cognitive impairment, which are major signs of dementia or Alzheimer’s Disease (AD)^7^. Under such stress, the brain undergoes a series of pathophysiological and mental alterations, especially in the hippocampus^8^. The hippocampus is a vulnerable and highly plastic region in the brain that plays a major role in long-term memory, memory consolidation, and emotions like fear, anxiety, and depression^9,10^. Its dysfunction is thus suggested to play a key role in anxiety and cognition.

Synaptic loss is a major correlate of cognitive impairment^11^. Previously, we reported impaired cognition and hippocampal GABAergic (gamma-aminobutyric acid) inhibitory synapses in animal models of transgenic AD and social isolation-induced anxiety^12,13^. The frequency, amplitude, and size of miniature inhibitory postsynaptic currents (mIPSCs) were reduced. At the same time, gephyrin, a postsynaptic GABA_A_R anchor protein that guides the formation and plasticity of GABAergic synapses, was reduced by 50%. However, this reduction of gephyrin levels only partially explains the GABAergic synapse dysfunction.

Glial cells in the brain closely associate with and physically support the structures of neuronal synapses^14^. Astrocytes are specialized glial cells that support the central nervous system by providing nutrients to neurons, maintaining extracellular environments, and providing structural support, including that of the synapse^15,16^. Astrocytes not only regulate metabolic supplies via blood vessels and neurons, but they also perform fine neurotransmission control by tightly enwrapping synapses and supporting appropriate signaling and insulation^17,18^. They form complex networks to support synaptic structure^19,20^, and there is increasing evidence for their involvement in complex behavioral functions including sleep, depression, and cognitive impairment^21^. Therefore, astrocyte could be a key target to improve cognitive function and mental wellbeing.

Astrocytes respond to stress and other insults in diverse manners to optimize their neuroprotective abilities. These changes vary with the severity of the damage and, depending on the response, can be either beneficial or detrimental^21,22^. For example, mild to moderate reactive astrocytes extend their cytoskeleton, form scars, and surround damaged tissues to protect healthy ones, while severe changes can cause undesirable upregulation or downregulation of gene expressions, cellular hypertrophy or atrophy, or scattered astrocyte proliferation^21^.

Dihydromyricetin [(2R,3R)-3,5,7-trihydroxy-2-(3,4,5-trihydroxyphenyl)-2,3-dihydrochromen-4-one] (DHM) is a flavonoid component isolated from the herbal plant *Ampelopsis grossedentata*. We previously reported that DHM acts as a positive allosteric modulator (PAM) of GABAergic transmission, and has anxiolytic activity, rescues GABA_A_ receptor (GABA_A_R) function, and restores gephyrin expression levels^13,23,24^. Thus, we hypothesize that astrocytes, synaptic supporters, are involved in this pathway. In this study, we utilized a social isolation-induced anxiety mice model^13^ to examine the effects of social isolation and DHM on cognition and plasticity of astrocytes in the hippocampus.

## Materials and methods

### Animals

All animal experiments were performed according to the protocols approved by the University of California (UCLA) and University of Southern California (USC) Institutional Animal Care and Use Committee (IACUC), and all methods were carried out in accordance with relevant guidelines, regulations, and recommendations, including the ARRIVE guidelines. Six-week-old male C57BL/6 mice (Charles River Laboratories, Hollister, CA) were housed in the vivarium under a 12 h light/dark cycle with direct bedding and free access to food and water. Animals were randomly assigned to experimental groups and habituated to the vivarium for 2 d before beginning experimentation. Two or three mice were allocated in each cage for group housing. For the social isolation groups, each mouse was separated into single cages wrapped with black plastic bags to prevent social interaction with other mice. We considered the mice to be in absolute social isolation as we singly housed them, wrapped their cages in opaque black bags, minimally handled them, and provided no environmental stimuli such as toys. We housed the mice in groups or social isolation for a period of four weeks. During the last two weeks, we orally administered either sucrose (vehicle) or DHM (2 mg/kg) daily. Tissue biochemical analyses were conducted at the University of Southern California (USC).

Groups were randomly separated as follows for a total of 4 weeks before sacrifice:

- G2 + Veh2: 2-week group housing plus 2-week group housing with vehicle (Veh) treatment
- G2 + D2: 2-week group housing plus 2-week group housing with DHM (D) treatment
- Iso2 + Veh2: 2-week social isolation (SoIso) plus 2-week SoIso with vehicle treatment
- Iso2 + D2: 2-week SoIso plus 2-week SoIso with DHM treatment

### Treatment preparation

DHM (HPLC purified ≥ 98%, Master Herbs Inc., Pomona, CA) 2 mg/kg was prepared in 3% agar cubes with 5% sucrose as described previously^13^. In short, 3% agar was dissolved in ~ 90 °C water, then mixed with DHM + 5% sucrose or 5% sucrose only until cooled and solidified. Treatment was prepared for the animals by cutting the agar into cubes of 0.5 × 0.5 × 0.5 cm and administering one cube per mouse. Both vehicle and DHM cubes were administrated orally once a day for the last two weeks during the dark period of the 12-h light/dark cycle with minimal disturbance to the mice. Complete consumption of the respective treatment was observed each day.

### Animal behavioral tests

Novel Object Recognition (NOR). We conducted the NOR test according to previous reports^24,25^. In short, the object recognition task is based on the cortex-dependent spontaneous tendency of rodents to explore a novel object for a longer period of time compared to a familiar one. On day one, the animals were familiarized with the empty open field for 5 min. On day two, they were subjected to a 5-min exploration session of two identical, symmetrically placed objects. 24 h later, the animals were subjected to a 3-min retention session where they were exposed to one familiar object from day two and one novel object. The times of exploration were recorded, and an object recognition index (ORI%) was calculated, such that $ORI\% = \frac{{t_{n} - t_{f} }}{{t_{n} + t_{f} }}$, where t_f_ and t_n_ represent times of exploring the familiar and novel objects, respectively.

Novel Context Recognition (NCR). We conducted the NCR test, which is dependent on the hippocampus, as described in previous reports^26^. The hippocampus plays a role in remembering a particular stimulus or object in a particular place^27^. In short, animals were exposed to two identical objects (i.e., two toy balls) in a round cage for 5 min and then to another two identical objects (i.e., two small cubes) in a rectangular cage for 5 min. After 24 h, animals were placed into either the round or the rectangular cage in which one of the objects was novel for that context (i.e., a toy ball and a small cube are placed into the round cage). The proportion of time spent investigating the novel “out of context” object versus the in-context object was calculated as a recognition index $RI\% = \left( {\frac{{t_{novel} }}{{t_{novel} + t_{sample} }}} \right) \times 100$ by a blinded scorer.

### Immunohistochemistry fluorescent staining, imaging and analysis

Mice were dissected the day following the end day of the 4-week experiments. Left brains were collected and fixed in 10% formaldehyde (FA, Sigma-Aldrich, USA) overnight at 4 °C. Brain tissues were then rinsed with 1X PBS (Sigma) and transferred to 30% sucrose solution (Sigma) at 4 °C for 3 days. Brains were washed in cold PBS and flash frozen in isopentane chilled with liquid nitrogen. Samples were immediately stored at -80℃. For cryosectioning, frozen brains were embedded in a mold with O.C.T. compound (Sakura, USA) on dry ice. 30 μm sagittal brain slices were sectioned at -20 °C with Microm HM525 Cryostat (Thermo, Waltham, MA) and transferred to SuperFrost microscope slides (VWR, USA). Slides were stored in − 80 °C until staining.

Antibodies were diluted in 1X PBS-Tween20 (PBS-T) + 10% BSA (w/v). Slides were incubated in a humidifier in mouse anti-mouse GFAP primary antibody (1:500, Cell Signaling #3670, RRID:AB_561049) for 2 days at 4 °C, and then in goat anti-mouse 550 nm fluorescent antibody (1:250, DyLight 550 #84,540, RRID:AB_10942171) overnight at 4 °C, protected from light. Stained slides were mounted with DAPI mounting medium (Abcam ab104139), coverslipped, and stored in 4 °C in dark until imaging.

All images were acquired on Zeiss LSM 880 confocal microscope (Carl Zeiss Microscopy, White Plains, NY) using Airyscan fast mode or Airyscan super-resolution mode. Images of the entire DG were acquired using 20X objective with 0.6X zoom to define the tile scan area and 2.0X zoom to define the laser settings and perform tile scanning. The following setups were used for the Z-stack scan: Z-range defined by GFAP (red) channel with 20X objective and 2.0X zoom, 2.5 µm interval, 1024 × 1024 frame size, and average acquisition at 8. After scanning, the images were processed by Zeiss Zen Black software for Airyscan Processing (3D), stitching, and maximum intensity projection.

Cell morphology was observed using 63X objective in Airyscan super-resolution mode. Single or near-single cells from similar portion on the DG area were selected using the 20X objective, snapped with frame size at 1024 × 1024, average acquisition at 1, and then switched to 63X to perform the Z-stack scan. The following setups were used for the Z-stack scan: Z-range defined by DAPI (blue) channel with 63X objective and 2.0X zoom, 0.2 µm interval, 1024 × 1024 frame size, and average acquisition at 1. After scanning, the images were processed by Zeiss Zen Black software for Airyscan Processing (3D), Gauss, and stack correction. 3D images were generated using 3D Surface option. All images were unified under same setups of X, Y, Z axis angles, threshold, light intensity ambient, specular light intensity, and surface shininess.

### Quantification and statistical analysis

All statistical analyses were performed using Prism v9.0.2. (GraphPad Software, Inc., La Jolla, CA, RRID:SCR_002798) or SigmaStat v3.5 (Systat Software, Inc.). Statistical details of the experiments can be found in the results and figures. Significance was defined as *P* ≤ 0.05. Analysis of cell density and size was performed by 3D object counter (FIJI plugin, RRID:SCR_002285). Cell counts per sample were normalized to the analyzed DG area (in µm^2^) and then multiplied by 1 × 10^6^ to show the number of astrocytes per 1000 µm^2^. Astrocyte complexity was analyzed by Sholl analysis (FIJI plugin).

### Significance statement

Social isolation induces stress and anxiety that lead to adverse changes in the brain. Proper brain function requires numerous essential factors, including specialized cells called astrocytes. In this paper, we show that mouse models of social isolation-induced anxiety exhibit deficits in memory and cognitive abilities, as well as plasticity changes in hippocampal astrocytes. We also show the therapeutic activity of dihydromyricetin, an herbal flavonoid component, to restore such abilities and reshape astrocytes. These findings are critical to demonstrate the impact of social isolation and stress on the brain and provide a potential preventative measure. Given the significant and continuing rise in anxiety, early and safe intervention is essential to prevent irreversible brain damage.

## Results

### Cognition and memory decline induced by social isolation is ameliorated by DHM

**Figure 1:**
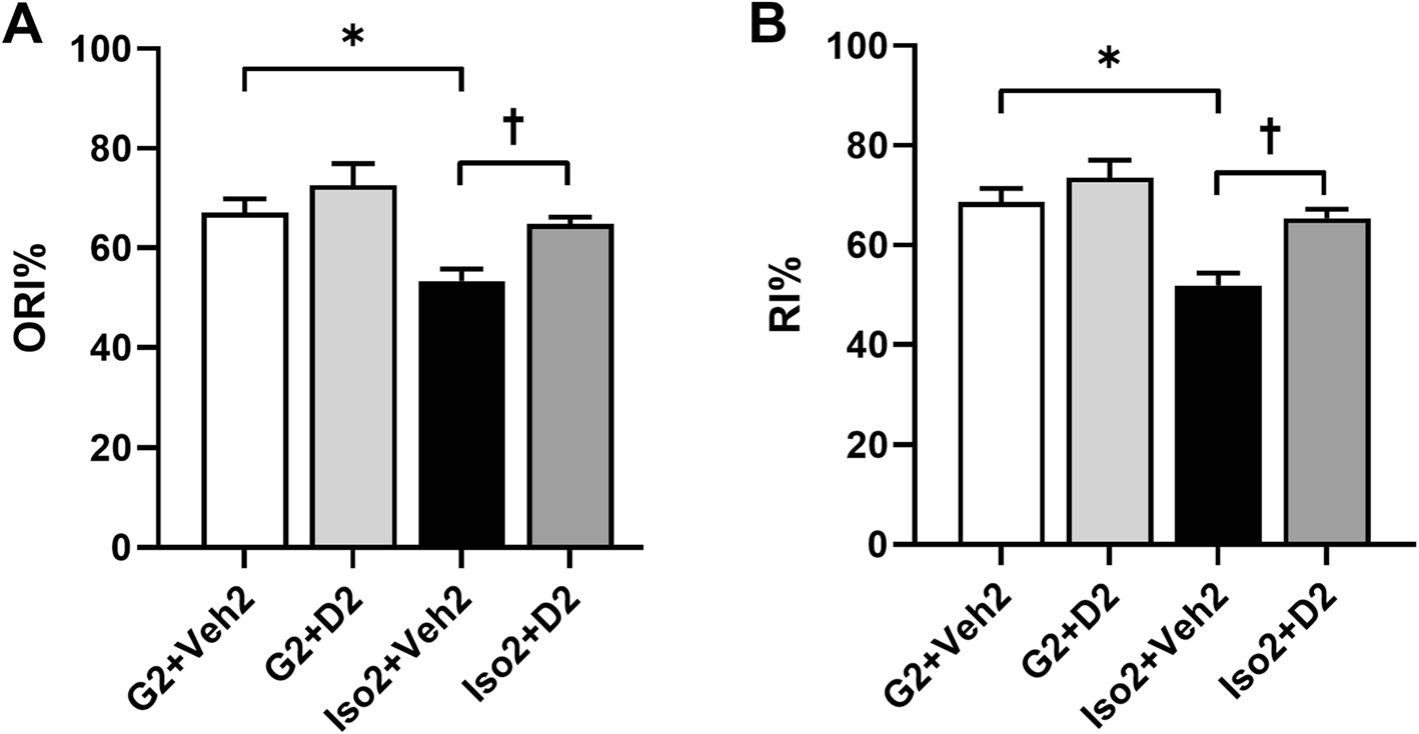
Cognition-related behaviors. (**A**) Novel object recognition. ORI = object recognition index. (**B**) Novel context recognition. RI = recognition index. G2 + Veh2: 2-week grouped plus 2-week grouped with vehicle treatment; G2 + D2: 2-week grouped plus 2-week grouped with DHM treatment; Iso2 + Veh2: 2-week SoIso plus 2-week SoIso with vehicle treatment; Iso2 + D2: 2-week SoIso plus 2-week SoIso with DHM administration. Bars represent mean ± SEM. Two-way ANOVA followed by multiple comparisons with Holm-Sidak method. *, *P* ≤ 0.05. †, *P* ≤ 0.05 vs Iso2 + D2.

Recognition memory is composed of at least two elements: the familiarity of items and the contextual information (spatial and/or temporal) in which the items were encountered^28^. In this study, we used two behavioral tests for evaluating both components of recognition memory: novel object recognition (NOR) test for familiarity of items, and novel context recognition (NCR) for contextual memory. Mice in social isolation exhibited worsened recognition memory (Fig. 1A). G2 + Veh2 mice spent more time exploring the novel objects with higher Object Recognition Index (ORI = 66.3 ± 4.7%) than Iso2 + Veh2 mice (ORI = 55.3 ± 4.1%) in NOR test (*P* = 0.0139, two-way ANOVA with multiple comparisons to the control, Holm-Sidak method). DHM improved object recognition memory of SoIso mice by roughly 9% (ORI = 64.2 ± 3.8%). Similarly, Recognition Index (RI) of NCR was calculated in every group of mice (Fig. 1B**)**. Compared with G2 + Veh2, Iso2 + Veh2 mice exhibited reduced RI (51.5 ± 6.5%, *P* = 0.002, two-way ANOVA with multiple comparisons to the control, Holm-Sidak method). DHM administration reversed the RI in Iso2 + D2 mice and showed substantial contextual memory improvement. These results indicate that daily oral administration of DHM restores memory and cognition in the SoIso mice.

### Social isolation and DHM induce changes in astrocytic density and size in the hippocampus

**Figure 2:**
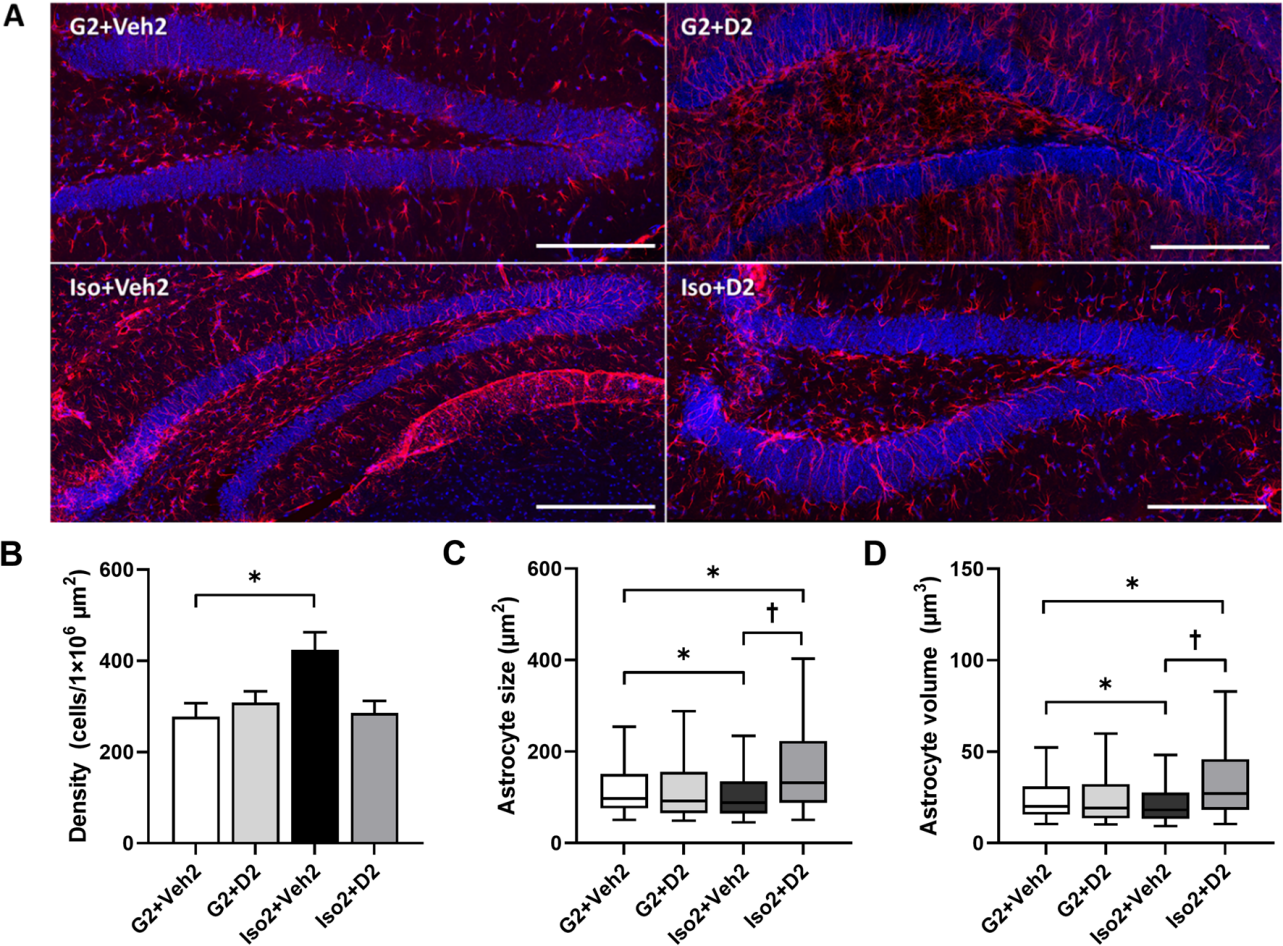
Astrocyte density and size in the dentate gyrus. (**A**) 20X Airyscan super resolution images of single or near-single astrocytes in the dentate gyrus of mice: blue = DAPI, nucleus; red = GFAP, astrocytes. Scale bar = 200 µm. (**B**) Number of astrocytes per sample normalized to DG area. Data shown as mean + SEM. Two-way ANOVA with multiple comparisons, Holm-Sidak method. Astrocyte size in µm^2^ (**C**) and µm^3^ (**D**). Data shown as box plot, where center line is the median, limits are the interquartile range (IQR), and whiskers are the minimum and maximum. Kruskal–Wallis one-way ANOVA on ranks with multiple comparisons, Dunn’s method. For all graphs, *N* = 3–4 mice analyzed per group, one section per mouse. G2 + Veh2: 2-week grouped plus 2-week grouped with vehicle treatment; G2 + D2: 2-week grouped plus 2-week grouped with DHM administration; Iso2 + Veh2: 2-week SoIso plus 2-week SoIso with vehicle treatment; Iso2 + D2: 2-week SoIso plus 2-week SoIso with DHM administration. *, *P* ≤ 0.05; †, *P* ≤ 0.05 vs Iso2 + D2; ‡, *P* ≤ 0.05 vs G2 + D2.

Next, we questioned whether social isolation induces astrocytic changes, as these changes are commonly observed in individuals with cognitive impairment, dementia, and AD^29^. To determine the effect of social isolation on astrocyte plasticity, we acquired sagittal mice brain images in the dentate gyrus (DG) using glial fibrillary acidic protein (GFAP) as the marker. Increased GFAP expression in astrocytes is commonly used as a marker for reactive astrocytes^30^. Our results show an increase in the number of GFAP-positive astrocytes in Iso2 + Veh2 compared to the control (G2 + Veh2) (424.2 vs 277.6 cells/1 × 10^6^ µm, *P* = 0.0117, two-way ANOVA with multiple comparisons to the control, Holm-Sidak method), which was reduced by DHM administration (Iso2 + D2) back to the control level (G2 + Veh2 [277.6] vs Iso2 + D2 [285.5] (Fig. 2A and B). Further, social isolation reduced astrocyte size compared to the control (Fig. 2C) (median 88.246 vs 97.223 µm^2^). Since the astrocyte size and volume are not normally distributed, we used Kruskal–Wallis one-way ANOVA with multiple comparisons, Dunn’s method to analyze them. The data are expressed as medians and percentiles with box plots. On the other hand, astrocyte sizes in DHM-administered mice (131.739 µm^2^) were larger compared to the control (DHM control (92.15 µm^2^), and SoIso mice), suggesting possible effects of DHM on mediating anti-inflammatory responses or recovery of astrocytic activities (Fig. 2C). These differences were mirrored in astrocyte volumes (Fig. 2D). G2 + Veh2 vs G2 + D2 showed no significant difference in size and volume.

### Social isolation and DHM induce morphological changes in the astrocytes of the hippocampus

**Figure 3:**
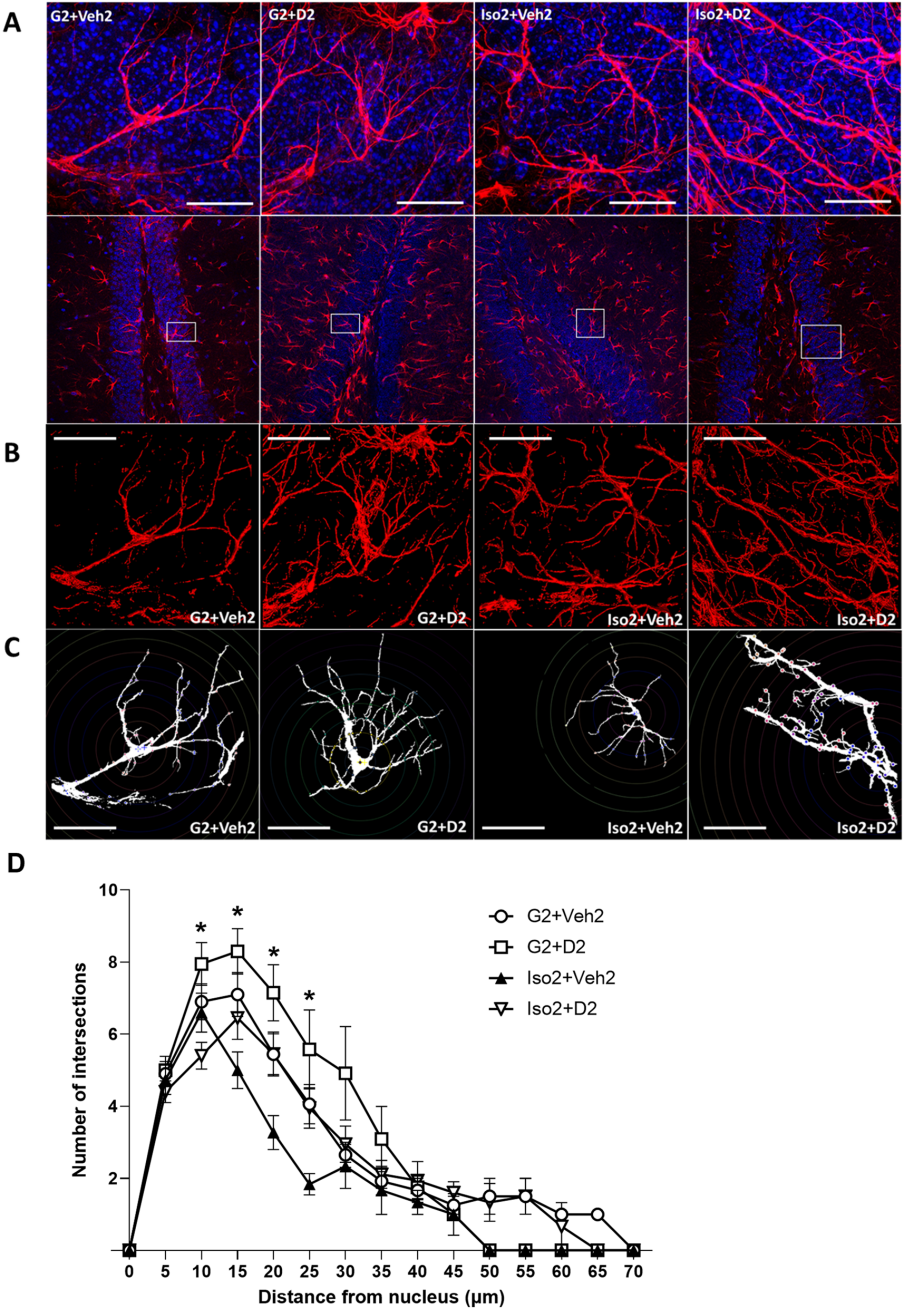
Astrocyte morphology in the dentate gyrus. (**A**) Upper: two-dimensional images of the astrocytes analyzed for morphology. Bottom: area of the DG containing the selected astrocyte. Blue = DAPI, nucleus; red = GFAP, astrocytes. Scale bar = 20 µm. (**B**) Three-dimensional images of astrocyte morphology. Scale bar = 20 µm. (**C**) Binary representation of astrocytes used for morphological complexity. Scale bar = 20 µm. (**D**) Quantification of morphological complexity (Sholl analysis). Two-way ANOVA with multiple comparisons, Holm-Sidak method. G2 + Veh2: 2-week grouped plus 2-week grouped with vehicle treatment; G2 + D2: 2-week grouped plus 2-week grouped with DHM administration; Iso2 + Veh2: 2-week SoIso plus 2-week SoIso with vehicle treatment; Iso2 + D2: 2-week SoIso plus 2-week SoIso with DHM administration. 5 astrocytes analyzed per mouse. *N* = 4 mice per group. *, *P* ≤ 0.05.

Astrocytes have dynamic and distinct morphological changes in response to the environment^22^. Consistent with Fig. 2, Iso2 + Veh2 showed more cells per area with reduction in branches, while DHM administration increased cell size with thicker, longer branches (Fig. 3). Three-dimensional Z-stack images in comparable areas of the DG for each group further demonstrate the increased activated astrocyte count in SoIso group (Fig. 3A, B, center) compared to the control (Fig. 3A, B, left), which was modulated by DHM (Fig. 3A, B, right). Astrocyte complexity as defined by Sholl analysis showed a decrease in Iso2 + Veh2 compared to the control (G2 + Veh2) at 10, 15, 20, and 25 µm^2^ from the nucleus (*P* = 0.0462, < 0.0001, 0.0009, and 0.0042, , respectively, two-way ANOVA with multiple comparisons to the control, Holm-Sidak method) and an increase with DHM administration at the same distances (Fig. 3C, D).

## Discussion

Loneliness serves as an early predictor for psychological problems like anxiety disorder that could further lead to suicidal thoughts and risk of later-life cognitive decline^31,32^. The COVID-19 pandemic and social isolation have amplified anxiety disorder prevalence that is likely to persist in the post-pandemic period. In this study, we found that social isolation worsened cognitive and memory abilities as it decreased ORI in NOR and RI in NCR tests, increased astrocyte density, reduced astrocyte size with shorter branches, and reduced morphological complexity in the hippocampus. DHM improved memory, cognition, and astrocyte plasticity in these mice. Our results indicate that social isolation leads to deficits in memory and cognition, as well as hippocampal astrocyte atrophy in mice models of social isolation-induced anxiety. Previously, we have shown that four weeks of social isolation considerably elevated anxious behaviors in mice, while two weeks of DHM administration reduced these behaviors^13^. In this study, we further illustrate the capability of DHM to improve cognitive loss induced by social isolation. Based on NCR and NOR, memory and cognitive abilities in socially isolated mice were restored by DHM administration, suggesting that DHM has neuroprotective properties that result in ameliorating these deficits (Fig. 1). Because the hippocampus is one of the first regions in the brain to suffer damage in mild cognitive impairment, including initial stages of AD^33,34^, we focused our imaging studies on the hippocampus.

Proper synapse distance and structure is essential for neurotransmission and thus essential for sharp cognition and memory. Astrocytes support synapse connection and thus are also essential players in cognition^17,18^. As Buss and colleagues (2021) have demonstrated, circuit organization breakdown was associated with diminished cognitive aging and reduction of DG synaptic input onto the CA3 pyramidal neurons^35^. Other studies have also found decreased synaptic density with decreased cognitive and memory abilities in individuals with AD^11,36,37^. Our present findings show decreased astrocyte complexity and size, in parallel with diminished cognitive and memory abilities in anxious mice (Figs. 1, 2C and 3), suggesting a reduction in astrocyte capability to support synapses. Further investigation is necessary to understand the relationship between astrocyte morphology and synaptic density/function, including studies involving synaptic markers.

The hippocampal astrocytes are mainly protoplasmic (highly branched), highly express GFAP, and are dynamic in changes related to immune response and synapse elimination^38^. Although GFAP expression is generally reduced in aged animals^39^, the connection between GFAP expression and astrocyte reactivity in AD is controversial. While many have found higher hippocampal GFAP levels with AD clinical progression, others have observed no difference between healthy and afflicted brains in humans^29^. The difference is likely attributed to both brain region^38^ and disease stage. At early clinical stages of transgenic AD mice models, astrocytes showed atrophy—reduced volume, surface area, and morphological complexity—and reduced GFAP expression, while disease progression corresponded to increased GFAP expression^22^. These observations were mirrored in post-mortem brains of AD patients, in addition to reduced astrocyte complexity and volume occupied by a single astrocyte^22,39^. Astrocytes in our socially isolated mice displayed increased cell density but reduction in size and morphological complexity (Figs. 2, 3), suggesting that social isolation can induce astrocyte atrophy, potentially disrupting synaptic support and neuroprotective abilities. While Hama et al. (2004) and Oyabu et al. (2020) have demonstrated that increased astrocytic density increased excitatory synaptic transmission in primary rat and mouse cultures^40,41^, our group previously found reductions in the inhibitory synaptic transmission (GABA_A_R) in mice brain slices^13^. Thus, in social isolation, increased astrocytic density may disrupt the balance of synaptic transmission due to increased excitatory transmission and decreased inhibitory transmission. This imbalance may lead to the nervous, restless, or aggressive behavior(s) observed in anxiety disorders^42^. Additional studies should examine the role of astrocytes in increasing or decreasing synaptic transmission, as well as upstream mechanisms of astrocytic proliferation and function.

Interestingly, while SoIso alone increased astrocyte density and reduced size, we found that SoIso-DHM administration did not alter astrocyte density (Fig. 2B) but exhibited larger astrocytes (Fig. 2C, D) compared to control. Furthermore, three-dimensional analysis showed that DHM-administered mice had longer, thicker astrocyte branches with morphological complexity greater than those in control, SoIso, and DHM control (Fig. 3). These findings are significant because after social isolation, DHM not only restored astrocytic density similar to that of control, but it also altered astrocyte morphology. These changes induced by DHM are similar to those shown in aged mice and rats that were exposed to environmental enrichments like toys, running wheels, and other mice^22^. These rodents showed increased morphological complexity and plasticity in astrocytes and improved cognitive abilities^22^. Our findings mirror these data, as DHM-administered mice exhibited recovery in cognitive and memory abilities (Fig. 1). Thus, social isolation induces symptoms of early aging in mice, while DHM recovers such symptoms, potentially by restoring astrocytic ability to support synapses.

In our previous studies with socially isolated mice, we reported that DHM reversed the reduced levels of adenine triphosphate (ATP) and gephyrin, a scaffolding protein that supports and stabilizes the clustering of post-synaptic GABA_A_ receptors^13^. We also recently demonstrated that DHM ameliorated GABA_A_R-mediated currents, microglia, and neuroinflammation in the same mice model^13,43^. GABA_A_Rs are highly expressed in astrocytes and allow them to sense and respond to their environment^44,45^. Receptor activation induces membrane depolarization—instead of hyperpolarization as in neurons—and increases intracellular Ca^2+^, stimulating the release of various signal molecules like ATP^46^. Neuroinflammation, however, has been shown to reduce astrocytic GABA_A_R expression and neurotransmission^47^. Collectively, these findings suggest that social isolation induces improper GABA_A_R clustering via gephyrin downregulation, subsequently reducing the ability of astrocytes to respond to their environment. Loss of this ability may induce astrocytic atrophy, which then leads to loss of homeostatic and/or synaptic functions^48^. The increased astrocytic density in the hippocampus could be a result of compensatory mechanisms, in which astrocytes migrate towards areas where synaptic transmission is reduced. We hypothesize that DHM modulates astrocyte plasticity and reverses/prevents these changes by promoting proper GABA_A_R clustering and functioning. Our hypothesis and upstream mechanism of gephyrin expression will be investigated in future studies.

In this study, we detected changes in memory, cognition, and hippocampal astrocytes in social isolation-induced anxiety mice models. These substantial changes within a short period of time suggest that repeated or prolonged anxiety and stress could lead to a greater long-term consequence, such as permanent brain damage, dementia, and Alzheimer’s Disease (AD). Furthermore, current anxiolytic medications are often not fully effective nor are readily available to individuals, so they have minimal ability to prevent these long-term consequences^42^. Novel drug development and marketing are timely and costly as well^49–51^, and thus cannot quickly address the sharp rise in anxiety, ASD, and declined mental wellbeing due to the pandemic. In contrast, DHM has a high potential to be developed as a quick and early intervention to anxiety, especially since the extensive process of developing therapeutics de novo is eliminated^50^.

This study is subject to a few limitations. First, anxiety prevalence and pathology has been shown to differ between sexes^52^, and our present study only included males. Thus, mirrored studies in female mice are ongoing. Because the astrocyte is a dense and compact area with overlapping branches, we could not obtain images for a true, single astrocyte. Morphological analysis, although threshold-based, may not be entirely accurate. Nonetheless, our findings shed light on the impact of social isolation on cognition and neuroprotective/astrocytic abilities.

Our findings of the adverse cognitive and cellular effects of social isolation suggest that social isolation can lead to early synaptic loss and aging signs. Further, we demonstrated the therapeutic activity of DHM to restore these cognitive damages and reshape astrocytes. Our results suggest astrocyte involvement in social isolation-induced cognitive impairment and anxiety and demonstrate the potential of astrocytes as a therapeutic target. Further, our study indicates DHM to be a promising candidate for early intervention against anxiety, cognitive impairment, and long-term risks of permanent brain damage. Prompt actions will delay brain aging, cognitive dysfunctions, and potential progression of severe diseases like dementia and AD. Early intervention is critical to prevent such irreversible outcomes.
